# Do Scholars-Turned-Businessmen Impact Green Innovation?

**DOI:** 10.3389/fpsyg.2022.920782

**Published:** 2022-06-20

**Authors:** Jing Zhao, Wanming Li, Qian Zhang

**Affiliations:** ^1^School of Economics and Management, Shihezi University, Shihezi, China; ^2^School of Economics and Management, Changji University, Changji, China

**Keywords:** seniors executives’ academic experience, green innovation, comprehensive competence, environmental concerns, managerial discretion

## Abstract

This study explores how the academic experience of executives affects green innovation. Using data on executive academic experience from a sample of Chinese listed companies, we explore the relationship between executive academic experience and green innovation using a combination of qualitative and quantitative methods. We find that executive academic experience has a positive impact on green innovation. We also investigate the moderating effect of managerial discretionary factors organizational slack, nature of property rights, and degree of market competition. The results show that organizational slack positively moderates the relationship between senior managers’ academic experience and green innovation, and this positive relationship is more significant in state-owned enterprises. The degree of market competition had a negative moderating effect on the positive relationship between academic experience of senior managers and green innovation. Improved general competence and concern for the environment are two possible mechanisms by which senior managers’ academic experience affects green innovation. Our findings suggest that academic experience of senior managers is an important factor for green innovation in emerging market firms.

## Introduction

In the context of the continuous increase in severe and extreme global weather and the novel Coronavirus pandemic, mankind has realized the urgency of environmental protection and the need to work together to address environmental problems. Environmental degradation is a bigger problem for emerging economies, in particular, because there is a huge environmental cost for their rapid economic development. Sustainable development is recognized by governments worldwide, and especially by emerging economies. Green innovation is a tool that can help firms achieve sustainable development and has attracted widespread attention in emerging economies (Steinmeier, 2016). Green innovation aims to both save resources and reduce enterprise pollution. It can improve an enterprise’s energy efficiency and reshape its image; therefore, it has become an active strategic behavior.

Managers’ decisions, which are influenced by their values and cognitive abilities, have an important impact on firms’ active strategic behavior. Imprinting theory believes that during the sensitive period, the external environment has an important and lasting influence on individuals’ value orientation and cognitive ability (Marquis and Tilcsik, 2013). Senior Executives’ specific experiences during sensitive times will influence their decision-making behavior. Existing literature focuses on senior executives’ military experience (Benmelech and Frydman, 2015), overseas experience (Giannetti et al., 2015), disaster experience (Bernile et al., 2017), and other influences on executive decision-making behavior. Literatures have confirmed that academic experience is an important sensitive period for individuals (Azoulay et al., 2017). Literature found that senior executives’ academic experience has an effect on enterprises’ major decisions such as innovation (Shen et al., 2020), investment and financing (Zhou et al., 2017), earnings management (Ma et al., 2019), and social responsibility (Shao et al., 2020). However, Scholars ignores on whether executive academic experience has an effect on corporate sustainability.

Therefore, we wanted to explore the relationship between senior managers’ academic experience and green innovation. Based on Imprinting theory, senior executives’ academic experience gives them ability and moral branding, as well as a high level of comprehensive ability and ethics. This will drive green innovation in companies. Because the particular circumstances faced by a company can influence the behavior of executives, we explore the boundary conditions of how executives’ academic experience affects green innovation based on the theory of managers’ discretion.

China is the largest emerging market country. It provides a good environment for our research. First, the Chinese government’s policy of encouraging people from universities and research institutions to participate in company operations has created a number of academic executives in Chinese enterprises. Second, the Chinese government has recognized the serious environmental problems caused by past economic development and has formulated many policies to support green innovation. On a practical level, it is also important to explore the relationship between executive academic experience and green innovation.

We collect data on executives’ academic experience from Chinese listed companies as a sample. We document a positive correlation between executive academic experience and corporate green innovation. We also explores the moderating effects of the organizational and external environments on executive academic experience and green innovation in the context of executive discretion. Finally, we argues that improving executives’ comprehensive ability and environmental concern are the two mechanisms.

The possible contributions of our study are as follows. First, it is a useful addition to the research on the economic gain of hiring managers with academic experience. Previous literatures confirmed that hiring senior executives with academic experience will affect enterprises’ investment and financing behavior (Zhou et al., 2017), innovation behavior (Shao et al., 2020), and information disclosure level (Francis et al., 2015). Our study proves that senior executives with academic experience can advance firm green innovation, which complements the abovementioned literature. Second, this study enriches the literature on the influencing factors of green innovation. Existing literature mainly focuses on the factors influencing green innovation from the external pressure and business strategy perspective. Few explore the influence of executives’ past experience on green innovation. From the perspective of executive academic experience, this study enriches the literature on the antecedents of green innovation. Third, we explore the moderating effect of executive discretion on the relationship between executive academic experience and firms’ green innovation and expand the boundary of the effect of executive characteristics on corporate strategy. Fourth, our research has implications for emerging economies on promoting the firm’s sustainable development by allowing developing countries formulate relevant policies to attract more talent with academic experience.

## Literature Review and Research Hypothesis

### The Determinants of Green Innovation

Green innovation refers to changes in production processes, new products, and management modes to reduce environmental and ecological pollution and improve energy efficiency. We divide the existing literature into three levels: external pressure, enterprise strategy, and manager characteristics and behavior. The external pressure mainly comes from the government’s environmental regulation and the environmental protection pressure from the stakeholders. Institutional theory holds that enterprises will try their best to meet the regulatory requirements of the outside world, such as the government, media, and various non-governmental organizations, to obtain legitimacy and maintain their survival and development (Berrone et al., 2013). In the face of increasingly stringent regulatory pressure from the government, enterprises initiate green innovation to reduce the litigation risk caused by environmental protection (Qi et al., 2018; Li and Xiao, 2020). Environmental demands and pressures from consumers (Chen et al., 2012; Lin et al., 2013), and even customers (Chen et al., 2017) and suppliers (Hart, 1995) influence enterprises’ green innovation (Berrone et al., 2013). Enterprise strategy research is mainly based on resource-based and natural resource-based perspectives. The natural foundation view holds that understanding the importance of environmental resources and consciously practicing environmental protection benefit enterprises in the fierce market competition. Literature shows that enterprises’ commitment to green innovation can reduce costs and gain the recognition of stakeholders. This enables enterprises to gain potential competitive advantages. Some literature also shows that the reputation gained by green innovation is conducive to the formation of unique proprietary assets (Juniati et al., 2019). The research on the management level is mainly based on Upper Echelons theory. Recent studies show Arrogance (Arena et al., 2018), time perception (Liao, 2016), hometown identity (Ren et al., 2021), and overseas experience (Wang et al., 2022) as executives’ personality attributes affect their participation in green innovation. These findings echo the core idea of Upper Echelons theory that senior executives’ experiences, values, and personalities influence their visions, alternative perceptions, interpretations, strategic choices, and results (Hambrick and Mason, 1984). However, although the academic experience may influence enterprises’ green innovation behavior by shaping executives’ cognitive ability and values, empirical evidence is lacking in this regard.

### The Effect of Senior Executives’ Academic Experience

Existing studies have proved the Upper Echelons theory. When managers make decisions in the face of complex internal and external environments, their decision-making process is not completely rational. Restricted by cognitive level and influenced by values, the personal characteristics of senior executives have a great influence on company’s business policymaking process (Schoar and Zuo, 2011). As one of the important professional experiences of senior executives, academic experience has a far-reaching influence on their cognitive ability, values, and innovation consciousness. Regarding cognitive ability and way of thinking, academic experience empowers executives with a more rigorous and objective way of thinking, which makes them behave more professionally and rationally, thus improving the quality of accounting information disclosed by the company (Francis et al., 2015). Senior executives’ objective and rational decision-making style can also improve the conservatism of accounting information, reduce earnings management, and thus reduce debt financing costs (Zhou et al., 2017). Senior executives are more conservative when influenced by academic experience. They rely more on professional knowledge to make judgments. Therefore, they will choose prudent financial policies and more physical investment, thus reducing the firm capitalization and stock price collapse risk (Du and Zhou, 2019; Guihua and Liuyun, 2021). Academic experience also has a far-reaching influence on the *sense of values* of senior executives and makes them more socially responsible. They pay attention to the relationship between enterprises and society, hope to contribute to society, and are more willing to fulfill their social responsibilities. Academic experience also makes senior executives highly innovative. Senior executives can have a natural interest in innovation and a high tolerance for innovation failure. The accumulation of academic experience also enables them to have enough knowledge and social capital to promote enterprise innovation (Schiuma et al., 2013). In sum, the factors influencing green innovation in the existing literature mainly focus on external pressure, enterprise strategy, and management personality. Previous studies have found that managers’ environmental awareness, beliefs, and attitudes toward environmental protection affect enterprises’ green innovation behavior profoundly (Chang, 2011). However, the literature does not pay enough attention to the effect of managers’ potential psychological mechanisms (past work experience) that influence decision-making on green innovation.

### Hypothesis Development

According to Imprinting theory (Marquis and Tilcsik, 2013), executives’ values and perceptions are largely influenced by their academic experience, and three conditions should be met in the process of branding individuals. First, there is a sensitive period in the process of personal growth. Second, individuals are greatly affected by the environment during the sensitive period. Finally, the behavioral characteristics formed during the sensitive period are persistent. Academic work can be regarded as a imprinting process, because academic experience is generally considered to be a critical sensitive period for individuals. Previous studies have suggested that early career is a critical sensitive period, because individuals are very vulnerable to the influence of the external environment during this period and need to deal with the associated uncertainties it brings (Gunz, 2007). Executives’ academic experiences typically occur at the early stages of their careers and therefore are critical and sensitive. Studies have confirmed that scientists’ research experience is a critical sensitive period (Azoulay et al., 2017). The third important factor in Imprinting is that the environment’s salient features can have a Major influence on the individual during sensitive periods. In such periods, individuals can have anxiety when facing the uncertain external environment (Schein, 1971); individuals will seek ways to reduce anxiety from peers, mentors and leaders (Gunz, 2007). Therefore, during the sensitive period, individuals will adopt cognitive models and behavioral norms adapted to the external environment (Azoulay et al., 2017). These cognitive models and behavioral norms influence their future behavior (Kacperczyk and Sutcliffe, 2009).

**FIGURE 1:**
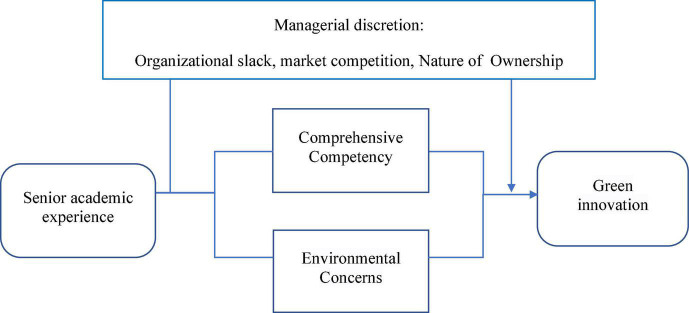
Theoretical framework diagram.

The nature of academic work is rigorous and highly competitive; individuals engaged in academic work must follow the mode and norms of academic work to adapt. A final characteristic of imprinting is its long-term persistence beyond the sensitive period. Research on scientists has found that even when individuals are in the early stages of their career, they will continue to maintain the beliefs, behaviors, and directions adopted in the formation stage (Azoulay et al., 2017). Green innovation is a corporate behavior with the dual characteristics of innovation and social responsibility. Inspired by this, we develop our hypotheses along two lines: (1) how academic experience influences executives’ comprehensive ability and (2) how academic experience influences executives’ attention to the environment. Then, we examine the factors that moderate the relationship between executive academic experience and green innovation from the perspective of executive discretion. The theoretical framework is shown in Figure 1.

#### Comprehensive Competency and Corporate Green Innovation

Academic experience brings an executive competency imprint that facilitates corporate green innovation. Compared to other green practices, green innovation is riskier, requires a greater knowledge of the firm, and has a longer payback period (Ahuja et al., 2008). Because of the high risk and long payback period, which can put pressure on investors in the short term, management will face questions from investors during the implementation of green innovation. Therefore, executives are required to have the commitment, creativity, and ability to lead a diverse group of people from different functional backgrounds and coordinate internal and external resources to promote green innovation. Green innovation also spills over in the sense that its results can easily be used by other firms; therefore, the development process is full of uncertainty (Chen, 2011). Due to its high degree of uncertainty, executives must be able to handle complex and dynamic information.

According to imprinting theory, an executive’s academic experience is a sensitive period, and the competencies developed during this period will have an impact on the executive’s future behavior. The extreme demand for innovation in academia has led executives to acquire a great deal of knowledge about innovation and to become familiar with the innovation process. As a result, they have a strong ability to innovate. Moreover, academic executives have the experience of investing significant resources in a questionable project at great professional risk (Jiang and Murphy, 2007), so they have a strong risk-taking ability. Finally, their academic experience gives them the ability to deal with complex and dynamic information, as they are used to using theory and logic to solve problems rather than prior experience when facing uncertain situations. In addition, being an academic gives executives the experience to announce their academic papers to the public and convince it to accept their academic views, as well as the ability to manage academic programs. Executives with academic experience thus have the ability to coordinate internal and external resources (Francis et al., 2015). All of these competencies are critical to green innovation. Thus, the competence imprint that academic experience brings to executives gives them a high level of overall competence that helps companies develop green innovation.

#### Environmental Concerns and Corporate Green Innovation

Based on imprinting theory, academic experiences can have a significant impact on executives’ environmental concerns. First, the main tasks of academic experience, teaching, research, and social service, emphasize individuals’ long-term social contributions (Francis et al., 2015). Second, Chinese Confucian culture has long required scholars to have a higher level of ethics and social responsibility. Confucianism emphasizes that scholars should “cultivate one’s moral character, cultivate one’s family, rule one’s country, and pacify the world,” and requires scholars to always remember their lifelong mission of dedication to society. Under these influences, Chinese scholars ingrain this sense of social responsibility in their hearts and form a fixed psychological value (Li and Liang, 2015), i.e., a moral imprint. Thus, when environmental protection has become a major social issue affecting the general public, executives with academic experience will be particularly concerned about how to reduce environmental hazards to enhance the general public’s interests. Third, many in the academic community view their reputation as fundamental to their survival in academia. As a result, executives with academic experience have an ethical imprint and value their reputation.

The Chinese government has incorporated environmental indicators into the assessment system of local governments, and companies that do not pay attention to environmental protection face huge environmental risks. Once a company’s environmental pollution is exposed, it will face enormous public pressure from the media and environmental organizations, as well as the operational risk of shutting down. Executives with academic experience usually also have certain social resources and high social visibility. When a company’s negative environmental pollution behavior is made public, executives will bear the brunt of public criticism and their reputation will be seriously damaged. Thus, the ethical imprint that academic experience brings to executives increases their environmental concern for the company and makes them more willing to advance green innovation.

Based on this analysis, we propose the main hypothesis:

H1: There is a positive relationship between senior executives’ academic experience and a firm’s green innovation.

### The Moderating Effect of Managerial Discretion

Suppose that senior executives’ academic experience affects enterprise innovation behavior. In such a case, what factors affect this relationship? Upper Echelons theory holds that executive discretion will influence the degree of executives’ influence on corporate strategy. Managerial discretion exists in relatively unconstrained areas and in areas where the means and consequences of actions are highly ambiguous (Hambrick and Mason, 1984). Executives can only apply their values, preferences, and experience to decision-making when the organization and environment give enterprises more choices. Therefore, managerial discretion becomes the most important boundary condition of Upper Echelons theory. Previous studies have proved organization and environment as the most important factors affecting managers’ discretion (Hambrick, 2007). This study explores the moderating effects of redundant enterprise resources (organizational factors), market competition, and enterprise nature (environmental factors) on senior executives’ academic experience and green innovation.

#### Redundant Resources of the Organization

Redundant resources refer to the resources existing in an organization that can be freely used and exceed the organization’s normal resource demand (Bourgeois, 1981; Qi et al., 2018; Amore and Bennedsen, 2016). Redundant resources can serve as a buffer for organizations to adapt to internal and external pressures. Simultaneously, redundant resources are also considered an effective resource that helps executives make strategic changes. According to previous studies, redundant resources help enterprises mitigate the external environment and provide the management with more freedom and control (Forbes and Milliken, 1999). For example, redundant resources can enable enterprises to undertake greater risks, improve product pricing, and gain more profits (Moses, 1992). Companies with redundant resources provide managers with more resources to accelerate the process of internationalization (Liu et al., 2011). Therefore, we predict that the richness of redundant resources will moderate the relationship between senior executives’ academic experience and green innovation. First, enterprises with redundant resources can mitigate the risks and uncertainties of green innovation activities and form a relatively loose innovation environment. Second, as a strategy for enterprises to cope with the external environment changes and obtain future core competitiveness, green innovation has a period of strategic opportunity, and redundant resources can improve the implementation efficiency of green innovation strategy. Finally, implementing a green innovation strategy requires enterprises to invest a large amount of capital in the early stage, and redundant resources can provide financial support for senior executives to effectively alleviate financing constraints in the process of green innovation. Therefore, enterprise redundant resources provide executives with more managerial freedom, thus moderating the relationship between senior executives’ academic experience and green innovation. Accordingly, the following hypothesis is put forth:

H2: The positive association between senior executives’ academic experience and firms’ green innovation is more pronounced for firms with redundant resources.

#### Market Competition

Previous studies show the heterogeneous enterprise environment as an important factor affecting managers’ discretion. Hambrick (2007) believes that market competition intensity affects the manager’s discretion. Fierce external competition threatens enterprises’ survival, reduces their profit margins, and forces them to invest more resources and energy to cope with external competition. As a result, the resources that managers can freely control will be reduced along with their discretion. Fierce external competition leads to reduced profit margins and a decline in investment capacity. Managers are forced to give up environmental investment in enterprises under the pressure of external survival. Studies have shown that when enterprises face increasingly fierce competition in the product market, they reduce their investment in CSR because of insufficient resources (Fernandez-Kranz and Santalo, 2010). Therefore, when an enterprise is in a highly competitive product market, it cannot provide sufficient resources and attention for its green innovation behavior because of insufficient resources and energy. On the contrary, when an enterprise is in an industry where competition is not fierce, it has control over the product market, has relatively rich resources, and managers’ discretion is also large. Executives with academic experience play a more important role in green innovation because companies can provide the resources and support needed for green innovation. Therefore, we predict that the relationship between senior executives’ academic experience and green innovation is more significant in industries with low market competition. Hence, the following hypothesis is proposed:

H3: The positive association between senior executives’ academic experience and a firm’s green innovation is more pronounced for firms in less competitive industries.

#### Nature of Ownership

In China, ownership structure influences the availability of enterprise resources and the manager’s power. The Chinese government strongly influences the allocation of resources needed by enterprises. Therefore, companies with closer government ties tend to have resource advantages. The natural close relationship between state-owned enterprises (SOEs) and the government enables them to have more resources than non-SOEs (Tan et al., 2007). For example, studies have found that SOEs are more likely to obtain external financing and government subsidies than non-SOEs (Bertrand and Schoar, 2003). Therefore, SOEs have more resources to develop green innovation. Additionally, managers of SOEs have more freedom and power than managers of non-SOEs. However, agency problems on property rights exist in SOEs, wherein the asset management departments usually exercise power on behalf of the state. Therefore, the absence of owners is a problem, and the lack of supervision in the practical sense centralizes the power in SOEs.

Additionally, the appointment authority of the senior executives and the board of directors of SOEs comes from the government. Moreover, the chairman and the senior executives are often government officials, limiting the effectiveness of the restriction of the board of directors on the senior executives. Therefore, senior executives have more power. The discretion of senior managers in SOEs is relatively large, which is conducive to the green innovation of senior executives with academic experience. The following hypothesis is proposed accordingly.

H4: The positive association between senior executives’ academic experience and a firm’s green innovation is more pronounced in SOEs.

To confirm our hypothesis, I will next test our proposed theoretical hypothesis by constructing an econometric model and using the corresponding econometric methods.

## Materials and Methods

### Sample Selection and Data

This study selected a sample of A-share listed companies in Shanghai and Shenzhen stock exchanges from 2009 to 2019. The executives’ data are hand-collected from their company’ annual reports and corporate top management team character database. Missing data was supplemented by additional information from public media, including Sina Financial, China economic network. Additionally, we deleted data on financial listed companies, insolvent companies, and companies with incomplete data. All continuous variables were tailed, and 23,071 observations were obtained to reduce the impact of extreme data values.

### Measurement

#### Measuring Academic Experience

According to prior research (Zhou et al., 2017), academic experience is defined as having been engaged in teaching or scientific research in universities or engaged in scientific research in professional non-profit scientific research institutions. This study uses the proportion of senior executives with academic experience to the total number of senior executives to measure the academic experience of senior executives.

#### Measuring Green Innovation

Based on the prior research (Amore and Bennedsen, 2016; Qi et al., 2018), this study uses the number of green patent applications of enterprises to measure green innovation. The process of green patent acquisition is as follows. The patent application and authorization of listed companies are inquired from the website of the State Intellectual Property Office of China’s official website (SIPO). We then use the SIPO “International Green Patent Classification List” to compare the number of green patents obtained by listed companies. We compare each patent with the codes in the International Patent Classification (IPC) Green List code base, and if the patent code matches one in the IPC patent code base, it is considered a green patent. Green patents are divided into three categories: “invention patents,” “utility models,” and “designs.” Invention patents can better reflect the quality of enterprise innovation. Therefore, we choose the number of green invention patents, plus one, and take the logarithm to measure the enterprise’s green innovation.

#### Control Variables

The control variables were selected according to previous studies examining the factors affecting green innovation (Ba et al., 2013). At the firm level, we control for firm size, which is the natural logarithm of the firm’s total assets, leverage, which is the natural logarithm of all liabilities divided by total assets, age, measured by the natural logarithm of the firm’s age plus one, return on net assets (Roa), measured by income divided by total assets, sales growth, measured by the growth rate of operating revenues. The level of cash holding (Cf) is measured by the amount of cash flow from operating activities divided by total assets at the end of the period. The company’s R&D investment (Rd) is measured by R&D expenditure divided by total assets. At the corporate governance level, we control for the proportion of independent directors (Indep) and the shareholding of the largest shareholder (Top1). Details of the measurement of all variables are provided in the variable definition table in Table 1.

**TABLE 1:** Variable definitions.

Variables	Variable name	Variable symbol	Variable definition
Dependent variable	Green Innovation	*GI*	Add 1 to the number of green patent applications, and then take the logarithm
Independent variables	Executives with academic experience	*ACADEMY*	The proportion of senior executives with academic experience in the total number of senior executives
Moderating variables	Nature of ownership	*SOEs*	If the state-owned enterprise is a state-owned holding enterprise, the value is 1; otherwise, the value is 0
	Redundant resources of the organization	*Slack*	Unprecipitated redundant resources = current assets/current liabilities Precipitated assets = (administration expenses + sales expenses)/sales expenses Potential redundant resources = total owners’ equity/total liabilities Redundant resources = mean after the normalization of the above three items
	Market concentration	*HHI*	HHI = sum(each company Revenue/Total income of the company’s industry)
Control variables	Ownership concentration	*Top1*	Shareholding ratio of the biggest shareholder
	Firm size	*Size*	*Ln*(Total assets of the company + 1)
	Asset-liability ratio	*Lev*	Total liabilities/total assets
	Time of establishment of the company	*Age*	*Ln*(corporate Listing time + 1)
	Return on equity	*Roa*	Net profit/total assets
	Company growth	*Growth*	Growth rate of operating revenue
	Cash holdings	*Cf*	Amount of cash flow from operating activities/Total assets at the end of the period
	R&D investment of listed companies	*RD*	R&D expenditure/total assets
	Proportion of independent directors	*Indep*	The proportion of independent directors in the total number of senior executives

*The above table provides a detailed description of the measures of the main variables.*

#### Moderators

The two moderating variables representing the firm level are SOE and redundant resources. SOE is a dummy variable that is 1 when the firm is a SOE and 0 otherwise.

Redundant resources are the firm’s idle assets (Singh, 1986). Our calculation process for redundant assets is: (1) Unsettled redundant resources are equal to current assets divided by current liabilities. (2) Sinking redundant resource assets are equal to the sum of overhead and selling expenses divided by selling expenses. (3) Potential redundant resources are equal to total owner’s equity divided by total liabilities. Finally, redundant resources are equal to the average value of unsinkable redundant resources, sinkable redundant resources assets, and potential redundant resources after normalization. This is because companies need sufficient resources to be invested within the company to carry out green innovation. These resources include both more flexible resources, such as capital, and more advanced resources such as management systems, so enterprises must always have unsinkable redundant resources. Potential redundant resources are a fundamental resource available to companies in the future. Studies have shown that potential redundant resources can help companies successfully implement innovation strategies. Therefore, in this manuscript, they are included in redundant assets.

The moderating variable representing the industry level is market competition. We use the Herfindahl index to measure a firm’s exposure to industry competition. The Herfindahl index is equal to the sum of the squared share of each firm’s sales to the sales of all firms in its industry. The smaller the Herfindahl index value, the more competitive the firm is in its industry.

### Empirical Model

To test H1, this study uses a model (Steinmeier, 2016) to test the effects of executives with academic experience on green innovation.


$$G\,I_{i,t}=\beta_{0}+\beta_{1}\,A\,C\,A\,D\,E\,M\,Y_{i,t}+\beta_{2}\,C\,o\,n\,t\,r\,o\,l_{i,t}+\sum I\,n\,d\,u\,s\,t\,r\,y$$

(1) +∑Y⁢e⁢a⁢r+ε

where *GI*_*i,t*,_ represents the green innovation of company *i* in year *t*, that is, the number of green patent applications. *ACADEMYi,t* represents the proportion of senior executives with academic experience in the total number of senior executives of *i* company in year *t*. We include all control variables *Control*_*i,t*,_. Year and Industry fixed effect are included.

In the following, we will verify the robustness of the main effects by transforming the measurement of independent and response variables, transforming the model, and removing part of the sample. Because of the possible endogeneity of the relationship between executive academic experience and green innovation, we will test it through the instrumental variables approach, PSM, and the Heckman model. Subsequently, we will also test the moderating effect proposed based on managerial discretion. Executive general competence and executive environmental concern, as two potential mechanisms we propose, will also be tested in the later section.

## Results

### Descriptive Statistics

Table 2 shows the results of descriptive statistics. The mean value of the dependent variable of green innovation *GI* is 0.241, and the standard deviation is 0.598. It shows that the green innovation of Chinese listed companies is low. The data is skewed to the right. The results are similar to those of Amore and Bennedsen (2016), Qi et al. (2018), and Li and Xiao (2020). The mean value of *academic* is 0.383, and the standard deviation is 0.182. This shows that 38.3% of the company executives have academic experience, showing that scholar executives occupy a certain share in company management. It also shows the practical significance of this research. In terms of control variables, the firms in our sample have an average firm size of 22.01 (which translates to total assets of RMB 2.84 billion), logarithm of firm age of 2.687 (which translates to a firm age of 8.457 years), sales growth of 0.226, ROA of 0.038, leverage of 0.408, cash-holding of 12.466, R&D investment of 0.021, board independence of 37.2%, and largest shareholder ownership of 34.5%. The results are similar to that of previous studies (Liao, 2016; Ren et al., 2021). Table 3 shows the results of the correlation coefficients between the main variables. The results show a significant positive correlation between the senior executives with academic experience and green innovation. This shows that increasing the proportion of scholar executives without controlling other variables promotes green innovation, consistent with Hypothesis 1. Additionally, the correlation coefficients of the independent, dependent, and control variables are less than 0.1, indicating no serious collinearity in the model.

**TABLE 2:** Descriptive statistics.

Variable	*N*	Mean	Std Dev	Min.	Median	Max.
*GI*	23071	0.241	0.598	0	0	3.332
*ACADEMIC*	23071	0.383	0.182	0	0.375	0.857
*Size*	23071	22.01	1.297	18.35	21.81	27.56
*Lev*	23071	0.408	0.211	0.0270	0.395	3.295
*Roa*	23071	0.0380	0.0730	−0.707	0.0400	0.313
*Growth*	23071	0.226	0.444	−0.560	0.106	4.497
*Cf*	23071	0.0440	0.0700	−0.289	0.0430	0.349
*Age*	23071	8.493	6.931	0	7	27
*RD*	23071	0.0210	0.0180	0	0.0170	0.115
*Top1*	23071	0.345	0.146	0.0800	0.326	0.758
Indepen	23071	0.375	0.0540	0.250	0.333	0.600

*The above table provides descriptive statistics on the main variables used in the empirical analysis. The sample used in this manuscript is 23,071 annual observations of companies listed in Chinese A-shares from 2009 to 2019. All continuous variables have undergone tailoring in order to mitigate the effect of outliers.*

**TABLE 3:** Correlation matrix.

	GI	Academic	Size	lev	Roa	Growth	Cf
*GI*	1						
*ACADEMIC*	0.045***	1					
*Size*	0.243***	−0.028***	1				
*Lev*	0.106***	−0.073***	0.493***	1			
*Roa*	0.027***	−0.0100	−0.020***	−0.362***	1		
*Growth*	−0.014**	0.027***	−0.096***	−0.186***	0.241***	1	
*Cf*	0.018***	−0.029***	0.063***	−0.149***	0.319***	−0.050***	1
*Age*	0.00900	−0.093***	0.426***	0.388***	−0.185***	−0.275***	−0.0100
*RD*	0.175***	0.121***	−0.213***	−0.224***	0.110***	−0.00400	0.098***
*Top1*	0.0110	−0.029***	0.179***	0.039***	0.119***	−0.00300	0.093***
*Indepen*	0.011*	0.144***	0.015**	−0.00800	−0.030***	−0.00500	−0.00700
	Age	RD	Top1	Indepen			
*Age*	1						
*RD*	−0.203***	1					
*Top1*	−0.076***	−0.104***	1				
*Indepen*	−0.028***	0.032***	0.047***	1			

The above table shows the results of Pearson correlation tests between the main variables. The results show that the correlations between executive academic experience, green innovation and most of the control variables are significant t-statistics ***, **, * denote significance levels of 0.01, 0.05 and 0.10, respectively.

Table 4 shows the results of the difference test between univariate groups. The sample is divided into a group with a higher proportion of *ACADEMIC* executives (higher than the mean of academic executives, *Academic Experience = 1*) and a lower proportion of academic executives (lower than the mean of *ACADEMIC* executives, *Academic Experience* = 0). The results of the inter-group mean and median difference test of *GI* show that the mean *GI* of the group with a higher proportion of senior scholars is 0.237, which is higher than the mean *GI* of 0.166 for the group with a lower proportion of senior scholars, and the result is significant at 1% level. The mean GI of the group with a higher proportion of senior scholars (0.237) was higher than that of the group with a lower proportion of senior scholars (0.166), and the result was significant at the 1% level. Similarly, the results of the inter-group median difference test showed that the median GI of the group with a higher proportion of scholar executives was significantly higher than that of the group with a higher proportion of scholar executives. The results show that academic executives have a significant effect on green innovation, and the main effect is valid.

**TABLE 4:** Univariate test.

Variate	Academic experience = 1			Academic experience = 0			Difference	
	*N*	Mean	Median	*N*	Mean	Median	Mean	Median
GI	15321	0.237	0.238	16013	0.166	0.166	0.131***	0.071***

The above table presents the results of the univariate mean and median tests. The sample was divided into two groups by the median value of the proportion of directors with executive academic experience. The group with higher proportion of directors with executive academic experience has Academic Experience equal to 1, otherwise it is 0. The results show that the level of green innovation (GI) is higher in the group with Academic Experience equal to 0. Significant t-statistics *** denote significance levels of 0.01 respectively.

### Executives With Academic Experience and Green Innovation

Table 5 shows the regression results of the main effects of top executives with academic experience on green innovation. Column (1) shows the regression results of only control industry and year dummy variables, not other control variables. Column (2) shows OLS results in which the control variables are added and the dummy variables for year and industry are controlled. The regression coefficients of independent variables with academic experience (*ACADEMIC*) are significantly positive at 1%, indicating that scholar executives are positively correlated with green innovation, and the main effect of H1 is verified.

**TABLE 5:** Association between executives with academic experience and green innovation.

	(1)	(2)
	*GI*	*GI*
*ACADEMIC*	0.145***	0.117***
	(7.59)	(5.01)
*Size*		0.174***
		(28.13)
*Lev*		0.045***
		(3.08)
*Roa*		0.046**
		(2.52)
*Growth*		−0.019***
		(−4.48)
*Cf*		0.113**
		(2.31)
*Age*		−0.008***
		(−11.53)
*RD*		0.314
		(0.94)
*Top1*		−0.001***
		(−4.17)
*Indepen*		−0.130*
		(−1.79)
*Constant*	−0.062***	−3.650***
	(−2.97)	(−27.01)
Year-FE	Yes	Yes
Industry-FE	Yes	Yes
*N*	23071	23071
AdjR^2^	0.114	0.168

The above table shows the regression results for model (1). We include industry and year fixed effects in the regressions. To control for the effects of heteroskedasticity and serial autocorrelation on the regression results, we control for the standard errors of clustering at the firm level. The t-statistic is shown in parentheses. ***, **, * denote significance levels of 0.01, 0.05 and 0.10, respectively.

### Robustness Test

#### Changing the Measurement of Green Innovation

This study sets green innovation as a dummy variable to verify the robustness of the regression results. When the enterprise has green innovation behavior (green patent), it is set to 1. When there is no green patent, it is set to 0. Column (1) in Table 6 shows that the coefficient of green innovation (*GI*) is significant at 10%, indicating that the basic regression results are still robust after replacing the measurement method of dependent variables.

**TABLE 6:** Robustness test results.

	(1)	(2)	(3)	(4)	(5)
	*GID*	*GI*	*L.GI*	*GI (TOBIT)*	*GI*
*ACADEMIC*	0.027*		0.078***	0.391***	0.159***
	(1.94)		(2.82)	(4.43)	(2.64)
*ACADEMICD*		0.061***			
		(2.85)			
*Size*	0.084***	0.162***	0.187***	0.539***	0.171***
	(31.11)	(30.27)	(24.47)	(38.68)	(17.53)
*Lev*	0.058***	0.075***	0.019	0.586***	0.163***
	(3.88)	(3.24)	(0.69)	(5.48)	(4.07)
*Roa*	0.146***	0.203***	0.049	1.032***	0.361***
	(4.13)	(4.01)	(0.78)	(3.75)	(5.16)
*Growth*	−0.012**	−0.022***	−0.098***	−0.072*	−0.022**
	(−2.28)	(−3.24)	(−7.80)	(−1.73)	(−1.98)
*Cf*	−0.026	−0.057	−0.111	−0.892***	−0.163
	(−0.70)	(−1.01)	(−1.52)	(−3.54)	(−1.61)
*Age*	−0.004***	−0.007***	−0.006***	−0.042***	−0.007***
	(−10.34)	(−9.73)	(−7.02)	(−15.05)	(−5.62)
*RD*	3.250***	5.367***	5.015***	24.609***	5.387***
	(17.96)	(17.68)	(13.08)	(37.63)	(11.58)
*Top1*	−0.001***	−0.001***	−0.001***	−0.006***	−0.220***
	(−4.04)	(−4.11)	(−3.43)	(−5.09)	(−4.31)
*Indepen*	−0.012	−0.034	0.058	−0.598**	−0.063
	(−0.26)	(−0.48)	(0.64)	(−2.08)	(−0.54)
*Constant*	−1.790***	−3.454***	−4.028***	−13.701***	−3.701***
	(−28.69)	(−29.42)	(−24.12)	(−46.77)	(−16.86)
Year-FE	Yes	Yes	Yes	NO	Yes
Industry-FE	Yes	Yes	Yes	NO	Yes
*N*	23071	23071	16426	23071	8080.000
*AdjR*	0.146	0.173	0.183	0.071	0.190

To test the robustness of the main regression, (1) we regress the dummy variable for green innovation on academic and firm-specific control variables, (2) replace academic experience with a dummy variable, (3) lag green innovation by one period, (4) replace the ols model with a tobit model, (5) remove heavy polluting firms. The regressions were rerun All variables are defined in Table 1. All continuous variables were screened at the 0.01 level. Regressions include industry and year fixed effects; robust standard errors based on heteroskedasticity and firm-level t(z) statistics are shown in parentheses. The t statistics in parentheses ***, **, * denote significance levels of 0.01, 0.05, and 0.10, respectively.

#### Reevaluating Executives With Academic Experience

This study uses the method of setting senior executives with academic experience as dummy variables to verify the robustness of the regression results. Column (2) in Table 6 shows a significant coefficient of academic (*ACADEMIC*) at 1%. Therefore, the basic regression result is stable after replacing the measurement method of academic executives.

#### Considering the Lag in Green Patents

There is a time interval between the application, approval, and application of green patents. Therefore, we tested H1 with the indicator of green innovation index lagging one period as the dependent variable. The regression results in Column (3) of Table 6 show that the coefficient of *GI* is significant at 1%, consistent with Hypothesis 1.

#### Transformation Model

As the green innovation application of listed companies is a counting variable, and there are many observation values of 0, this study selects the Tobit model to verify the stability of the main hypothesis. Column (4) of Table 6 shows that the coefficient of *ACADEMIC* is significant at the 1% level, indicating the robustness of the main effect.

#### Removing Samples From Heavy Pollution Industries

Because of heavy pollution, industry is greatly affected by environmental regulation and other related policies. Therefore, to eliminate the mandatory effect of environmental protection policies on green innovation, this study deletes heavy pollution industry data and then tests the effect of academic executives on green innovation. Column (5) of Table 6 shows a significant coefficient of *ACADEMIC* at the 1% level after deleting heavy pollution industries, which indicates that the main effect is robust after excluding policy influence.

### Endogeneity Test

Because companies that engage in green innovation are more likely to be large-scale, have higher efficiency, and focus on CSR, they are more likely to attract executives with academic experience. The main effect mentioned above may be endogenous of bidirectional causality. Therefore, to weaken the influence of endogenous problems on the main effect, this study uses instrument variables (IV) estimation, Heckman two-stage, and propensity score matching (PSM) method to verify the effect.

#### The Instrumental Variable

The key of the IV method is finding the appropriate instrumental variable. This study selects the number of higher education institutions in the provinces where listed companies are registered as an instrumental variable. Higher education institutions in the province where the employer’s company is registered result in more communication opportunities between the university and the enterprise. Therefore, the enterprise will hire senior executives with academic experience. However, there is no evidence that the number of higher education institutions in the province where the parent company is incorporated is significantly related to green innovation. Column (1) of Table 7 shows the results of the first stage regression, where the coefficient of IV is significantly positive at the 1% level. This indicates that the instrumental variable is highly correlated with the likelihood that the firm has executives with academic experience. Column (2) of Table 7 shows the results of the second stage regression of the IV method. The results show that ACADEMIC is significantly positive at the 1% level, confirming that the main effect remains unchanged after controlling for endogeneity.

**TABLE 7:** Endogeneity test.

	(1)	(2)	(3)	(4)	(5)
	
	*Instrumental variable method (2SLS)*		*Hackman*		*PSM*
	*First stage*	*Second stage*	*First stage*	*Second stage*	*Matched sample*
*Iv*	5.016***		1.317***	*	
	(7.480)		(3.21)		
*ACADEMIC*		0.226***		0.219***	0.223***
		(4.25)		(4.29)	(4.36)
*IMR*				5.845***	
				(6.05)	
*Size*	0.002***	0.188***	0.296***	1.209***	0.188***
	(2.84)	(19.47)	(29.46)	(6.97)	(19.39)
*Lev*	0.008***	0.045*	0.339***	−1.447***	0.045*
	(2.15)	(1.86)	(5.23)	(−5.82)	(1.85)
*Roa*	0.011***	0.052*	0.493***	0.116***	0.052*
	(2.56)	(1.72)	(2.99)	(2.86)	(1.71)
*Growth*	0.001	−0.024***	−0.035	−0.028***	−0.024***
	(0.61)	(−4.34)	(−1.34)	(−4.92)	(−4.33)
*Cf*	0.009	0.182**	−0.509***	0.068	0.182**
	(0.92)	(2.39)	(−3.26)	(0.87)	(2.38)
*Age*	−0.001*	−0.007***	−0.025***	−0.142***	−0.007***
	(−1.96)	(−6.48)	(−14.04)	(−6.25)	(−6.46)
*RD*	0.002*	0.129	16.865***	0.128	0.129
	(0.35)	(0.71)	(31.68)	(0.70)	(0.70)
*Top1*	0.007*	−0.159***	−0.375***	−0.179***	−0.159***
	(1.98)	(−3.62)	(−5.16)	(−4.09)	(−3.60)
*Indepen*	0.109***	−0.164	−0.196	−0.236**	−0.164
	(3.98)	(−1.63)	(−1.03)	(−2.33)	(−1.61)
*Constant*	−8.089***	−3.942***	−8.089***	−34.003***	−3.940***
	(−32.79)	(−18.33)	(−32.79)	(−6.71)	(−18.26)
*N*	23071	23071	23071	23071	11290
*AdjR* ^2^	0.146	0.178	0.135	0.185	0.178

The above table gives the results of the tests dealing with endogeneity. Columns (1) and (2) show the results of the test for the instrumental variable method. Column (1) shows the results of the regression with the number of higher education institutions in the company’s location as an instrumental variable. Column (2) shows the results of the second stage regression for the IV method. Columns (3) and (4) show the results of the heckman two-stage method. Column (3) shows the results of the regression with the number of higher education institutions in the company’s location as the instrumental variable. Column (4) shows the inverse Nielsen ratio calculated in the second stage. Column (5) shows the results of re-running the regression on the sample after the sample was psm matched. The t statistics in parentheses, ***, **, * denote significance levels of 0.01, 0.05, and 0.10, respectively.

#### Heckman Two-Stage Method

Although, our study confirms a positive correlation between senior executives’ academic experience and green innovation, this result may be affected by self-selection bias because companies with certain characteristics are more likely to hire executives with academic experience and tend to have more green innovation. In other words, the hiring decision is not a random choice, which can be conducive to self-selection bias. To mitigate this potential problem, we apply the Heckman selection model.

In the first stage, all control variables in Model 1 are included in the logit model to estimate the likelihood of a firm hiring executives with academic experience. We use the number of higher education institutions in the province where the firm is located as an instrumental variable. Because it has been explained previously, this variable satisfies the exogeneity requirement of the instrumental variable.

We report the results in Table 7. Column (3) shows the regression results of the number of higher education institutions in the province where listed companies are registered as the IV in the first stage. The results show that the IV is significant at the 1% level. Then the inverse Mills ratio (IMR) (inverse Niersby) calculated in the first stage was added to the second stage for regression. Column (4) in Table 7 shows that the coefficient of the IMR is significant, and the coefficient of *ACADEMIC* is significantly positive at 1%, indicating that the conclusion of the main effect is robust after controlling the self-selection problem of samples.

#### Propensity Score Matching Method

**FIGURE 2:**
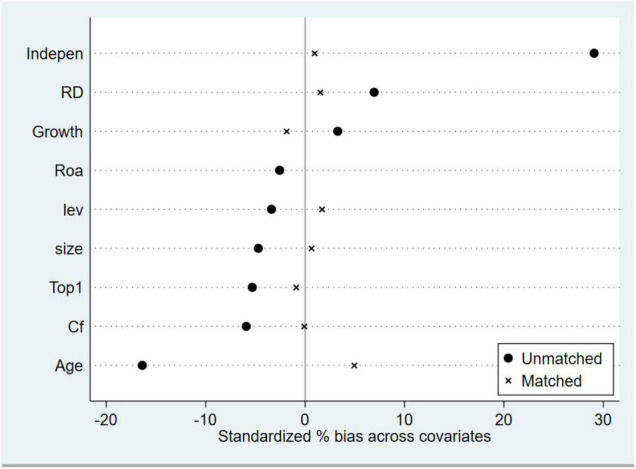
Balanced test results. We subjected the control and treatment crude samples that had undergone psm to a balance test. The standard deviations of the control variables of the samples of the control and treatment groups were basically within 10%, indicating that the sample characteristics of the control and treatment groups were basically the same after the psm.

Firms with a high proportion of academic executives differ from firms with a low proportion on many dimensions. To reduce the interference of such differences in the stability of the main effects, we use PSM to test this. The sample firms were first divided into an experimental group with a high percentage of academic executives and a control group with a low percentage of academic executives by the median percentage of academic executives. The group with a lower percentage of academic executives was then set to 1 and the rest to 0. Next, we regressed all control variables on executive academic experience using a logit model and estimated propensity scores for firms where executives had academic experience. Next, we matched each treatment-group firm to the control-group firm with the closest propensity score at a ratio of 1:3. The results showed that the control variables are not significantly different between control group and treatment group firms (Figure 2). The matched samples were then regressed. Column (6) of Table 7 shows that the coefficient on ACADEMIC is significantly positive at the 1% level after matching the differences between the propensity scores and the control sample at the firm level, a result that is consistent with the main effects test.

### Moderating Effects

This section examines the moderating effect of managers’ freedom on the relationship between senior executives’ academic experience and green innovation. We first investigate the moderating effect of organizational slack on the relationship between senior executives’ academic experience and green innovation (H2). Column (1) in Table 8 shows that when green innovation is taken as a dependent variable, the coefficient of executive academic experience* organizational slack is 0.001 (*t* = 3.16). This indicates that when organizational slack positively affects the relationship between executive academic experience and green innovation, it is consistent with H2. Next, we study how the degree of market competition affects the relationship between senior executives’ academic experience and green innovation. This result is shown in Column (2) of Table 8. The regression coefficient of the degree of market competition of senior executives’ academic experience is positive at 1% and statistically significant (2.576, *t* = 2.59). The results show that when the market competition is not intense, the positive relationship between senior executives’ academic experience and green innovation is more significant, consistent with H3. Finally, we found that the coefficient of Academic*SOE was significant and positive at 1% (0.188, *t* = 3.37). This result indicates that the positive relationship between senior executives’ academic experience and green innovation in SEOs is more significant.

**TABLE 8:** Moderating effects.

	(1)	(2)	(3)
	*GI*	*GI*	*GI*
*Academic*	0.121***	0.112***	0.058**
	(5.16)	(3.98)	(2.37)
Academic* Slack	0.001***	–	
	(3.16)		
*Slack*	0.000***		
	(3.18)		
*HHI*		1.336***	
		(3.42)	
Academic*HHI		2.576***	
		(2.59)	
*state*			0.024
			(1.07)
Academic* SOEs			0.188***
			(3.37)
*Size*	0.175***	0.188***	0.169***
	(28.20)	(25.09)	(27.50)
*Lev*	0.045***	0.049**	0.036**
	(3.06)	(2.49)	(2.41)
*Roa*	0.046**	0.048*	0.034*
	(2.49)	(1.87)	(1.86)
*Growth*	−0.020***	−0.016***	−0.016***
	(−5.76)	(−3.59)	(−4.06)
*Cf*	0.116**	0.006	0.118**
	(2.34)	(0.10)	(2.41)
*Age*	−0.008***	−0.007***	−0.011***
	(−11.69)	(−7.98)	(−14.21)
*RD*	0.314	4.745***	0.317
	(0.94)	(12.41)	(0.95)
*Top1*	−0.125***	−0.159***	−0.177***
	(−4.13)	(−4.51)	(−5.78)
*Indepen*	−0.129*	−0.159*	−0.100
	(−1.77)	(−1.87)	(−1.38)
*Constant*	−3.682***	−3.978***	−3.563***
	(−27.09)	(−24.13)	(−26.48)
Year-FE	Yes	Yes	Yes
Industry-FE	Yes	Yes	Yes
*N*	22,991	16,202	23,069
Year/Industry	Yes	Yes	Yes
AdjR^2^	0.168	0.192	0.171

The above table shows the regression results of the moderating effects of incorporating redundant resources, the nature of ownership, and the degree and the degree of market competition. All continuous variables were winsorized at the 0.01 level. Regressions include industry and year fixed effects; robust standard errors based on heteroskedasticity and firm-level t(z) statistics are shown in parentheses. The t statistics in parentheses. ***, **, * denote significance levels of 0.01, 0.05, and 0.10, respectively.

### Potential Mechanisms

This section explores two potential mechanisms of executive academic experience on green innovation: improving management integration and environmental concern.

#### Comprehensive Competence

The theoretical analysis of this study illustrates that one of the potential mechanisms by which executive academic experience promotes green innovation is improving executives’ comprehensive ability. We predict that senior executives with academic experience can significantly improve the comprehensive ability of other senior executives. If the results are as expected, green innovation is more effective in diversifying companies. As the diversification strategy of an enterprise represents the complexity of the company’s business, the ability of senior executives to deal with dynamic and complex affairs is expected to be higher. According to the literature Hann et al. (2013), business units were used as diversified indicator agents to test the hypothesis (Hann et al., 2013). If a company has more than one division, we set diversity to 1, which means the company has diversified operations, otherwise it is 0. This study divides the enterprises into two sub-samples with and without diversified behaviors for testing. The inspection results are reported in Table 9. The difference of the test coefficient indicates that the positive effect of senior executives’ academic experience on green innovation is more significant in diversified enterprises. The result is consistent with our expectations.

**TABLE 9:** Mechanism test results (comprehensive ability).

	(1)	(2)
	GI (div = 1)	GI (div = 0)
*Academic*	0.131***	0.077**
	(3.56)	(2.47)
*Size*	0.202***	0.156***
	(18.82)	(19.20)
*Lev*	0.157***	−0.038
	(5.01)	(−1.32)
*Roa*	0.182***	−0.022
	(4.49)	(−0.65)
*Growth*	−0.007	−0.020***
	(−0.63)	(−4.18)
*Cf*	−0.262***	0.225***
	(−3.08)	(3.57)
*Age*	−0.007***	−0.008***
	(−5.25)	(−8.47)
*RD*	4.243***	0.125
	(9.22)	(0.75)
*Top1*	−0.238***	−0.109***
	(−5.22)	(−2.61)
*Indepen*	0.017	−0.117
	(0.15)	(−1.19)
*_cons*	−4.296***	−3.281***
	(−18.04)	(−19.14)
*Year-FE*	*Yes*	*Yes*
*Industry-FE*	*Yes*	*Yes*
*N*	12471	9434
*AdjR* ^2^	0.191	0.178

First, we divide the sample into firms with diversification behavior and firms without diversification behavior. Secondly in both subsamples we regress green innovation on academic and control variables. Regressions include industry and year fixed effects; robust standard errors based on heteroskedasticity and firm-level. t-statistics are shown in parentheses. The t statistics in parentheses. ***, ** denote significance levels of 0.01, 0.05 respectively.

#### Environmental Concerns

Another mechanism discussed in the hypothetical development section is environmental concern. Enterprises that pass ISO14001 environmental certification have higher requirements for environmental protection (Potoski and Prakash, 2005). Therefore, we predict that senior executives with academic experience pay more attention to the environment, thus promoting green innovation. We tested whether companies with senior executives with academic experience are more likely to pass ISO14001 environmental certification. Therefore, the sum-samples may be enterprises that have passed ISO14001 environmental certification and those that have not. Table 10 shows the test results. They show that the regression coefficient of senior executives’ academic experience on green innovation in enterprises that have passed ISO14001 environmental certification is significantly greater than in enterprises without environmental certification. The difference in the test coefficient indicates that the positive effect of senior executives’ academic experience on green enterprise innovation is more significant in enterprises that have passed ISO14001 environmental certification. This is consistent with Hypothesis 1. Further, public relations academic experience increases the output of green innovation by improving enterprises’ environmental attention.

**TABLE 10:** Mechanism test results (environmental concerns).

	(1)	(2)
	GI (IsPassISO14001 = 0)	GI (IsPassISO14001 = 1)
*Academic*	0.070***	0.192***
	(2.63)	(3.18)
*Size*	0.155***	0.257***
	(22.18)	(15.05)
*Lev*	0.052***	0.004
	(3.39)	(0.06)
*Roa*	0.058***	0.264**
	(3.04)	(2.12)
*Growth*	−0.016***	−0.053***
	(−3.91)	(−3.53)
*Cf*	0.083	−0.142
	(1.55)	(−0.90)
*Age*	−0.008***	−0.010***
	(−9.75)	(−4.91)
*RD*	0.181	8.273***
	(0.84)	(9.29)
*Top1*	−0.123***	−0.065
	(−3.48)	(−0.87)
*Indepen*	−0.036	−0.077
	(−0.43)	(−0.42)
*_cons*	−3.211***	−5.531***
	(−16.63)	(−12.96)
*Year-FE*	*Yes*	*Yes*
*Industry-FE*	*Yes*	*Yes*
*N*	15911	4939.
AdjR^2^	0.159	0.226

First, we divided the sample into companies that passed ISO14001 and companies that did not pass ISO14001. Second in both subsamples, we regress green innovation on academic and control variables. Regressions include industry and year fixed effects; robust standard errors based on heteroskedasticity and firm-level t(z) statistics are shown in parentheses. ***, ** denote significance levels of 0.01, 0.05 respectively.

## Discussion

We investigated the impact of executives’ academic experience on corporate green innovation. Using data from Chinese listed companies from 2010 to 2019 as a sample, this manuscript finds that executives with academic experience can significantly promote green innovation in firms by collecting data on their academic experience. Consistent with managerial discretion theory, the positive relationship between executive academic experience and green innovation is more significant when executives are given more resources and latitude. For example, we found that the positive relationship is more significant in SOEs, which have more redundant resources and lower levels of market competition. To demonstrate the robustness of our results, we used a series of tests such as substitution of strain variables, transformation models, Heckman, and PSM. The results show that the positive relationship between executive academic experience and green innovation is robust. In addition, the test results indicate that executives with academic experience have stronger general competence and environmental concern compared to other executives, as an important mechanism to promote green innovation in companies.

This manuscript examines the influence of executives’ past experiences on corporate green innovation based on imprinting theory. This differs from previous investigations of the influences on green innovation in terms of external pressures, organizational strategies, and managers’ direct attitudes toward environmental protection. Our findings suggest that executives’ academic experience helps to improve green innovation in firms. Because academic experience empowers executives with the intrinsic moral motivation and ability to implement green innovation. Our study enriches the literature in the area of the relationship between managerial behavior and corporate sustainability.

The practical significance of this manuscript is as follows: First, our research found that executives’ academic experience can promote enterprises carrying out green development. Therefore, the government should introduce corresponding policies to provide resources for university-enterprise cooperation and establish institutional guarantee for university teachers to serve enterprises, so that enterprises can make full use of universities’ talent resources and receive intellectual support for green development. For enterprises, this is an opportunity to properly introduce executives with academic experience to establish talent guarantee for enterprises to continuously promote green innovation. Second, our study found that executives’ strong comprehensive ability and high environmental concern are important mechanisms to promote green innovation in enterprises. In view of this, companies should focus on selecting executives with strong overall ability and high environmental concern to help better promote green innovation. Third, executives with academic experience can better promote green innovation when companies have more redundant resources. Therefore, the government should consider providing appropriate subsidies to incentivize green innovation in resource-poor firms. Fourth, while the effect of academic experience on green innovation is not significant in non-SOEs, the state may consider introducing corresponding policies to support green innovation behavior in non-SOEs since SOEs have more abundant resources to facilitate executives with academic experience to promote green innovation. Fifth, our study confirms that executives with academic experience are more willing to engage in green innovation in industries with lower levels of market competition. The government should introduce policies to deregulate the industry to guide enterprises into new investment areas and moderately reduce the intensity of market competition so that they can invest resources in green innovation.

The impact on green innovation can vary as individual members of the executive team have a significantly different impact on the organization, and core members of the executive team such as the CEO, CFO, and CTO have more resources at their disposal than regular executives. The next step is to explore the different impacts of core executive members (CEO, CFO, and CTO) on green innovation.

## Data Availability Statement

The original contributions presented in this study are included in the article/supplementary material, further inquiries can be directed to the corresponding author.

## Author Contributions

JZ: conceptualization, methodology, software, and writing – review and editing. WL: conceptualization. QZ: writing – review and editing. All authors contributed to the article and approved the submitted version.

## Conflict of Interest

The authors declare that the research was conducted in the absence of any commercial or financial relationships that could be construed as a potential conflict of interest.

## Publisher’s Note

All claims expressed in this article are solely those of the authors and do not necessarily represent those of their affiliated organizations, or those of the publisher, the editors and the reviewers. Any product that may be evaluated in this article, or claim that may be made by its manufacturer, is not guaranteed or endorsed by the publisher.
